# Psychological Interventions Added to Standard Care Improve Pain and Function Outcomes in Knee Osteoarthritis: A Systematic Review and Meta‐Analysis

**DOI:** 10.1002/msc.70141

**Published:** 2025-06-18

**Authors:** Tim Phelps, Jonathan Gilby, Joanne Hosking, Jonathon Gill

**Affiliations:** ^1^ Cornwall Partnership NHS Foundation Trust Bodmin UK; ^2^ School of Health Professions, Faculty of Health University of Plymouth Plymouth UK; ^3^ Livewell Southwest CIC Plymouth UK; ^4^ Peninsula Medical School University of Plymouth Plymouth UK; ^5^ Somerset NHS Foundation Trust Taunton UK

**Keywords:** cognitive behavioural therapy, function, knee osteoarthritis, pain, pain coping skills training, systematic review

## Abstract

**Objective:**

Knee osteoarthritis is a primary cause of disability across the world and current standard care fails to address all biopsychosocial contributions to pain. The current review aims to evaluate randomised controlled trials examining the effect of adding cognitive behavioural therapy or pain coping skills training to standard care on pain and function outcomes for individuals with knee osteoarthritis.

**Data sources:**

Systematic searches were conducted of CINAHL, EMBASE (OVID), Medline (EBSCO) and PsycINFO databases until July 2024 with no date restrictions.

**Methods:**

This systematic review followed the preferred reporting items for systematic reviews and meta‐analyses (PRISMA) guidelines. Risk of bias was assessed using the Risk of bias 2 tool. Meta‐analysis using a random‐effects model was carried out using the ​Statistical Package for the Social Sciences, and effect sizes from standardised mean differences were calculated using Cohen's d statistic. Heterogeneity was assessed using I‐squared and Tau‐squared tests.

**Results:**

Four randomised controlled trials met eligibility criteria (*n* = 628, mean age 62.91), demonstrating a low risk of bias. The addition of cognitive behavioural therapy or pain coping skills training to standard care for knee osteoarthritis produced statistically significant changes in standardised mean differences (*p* < 0.001), showing small to medium effect sizes in pain (0.488) and function (0.340) between 3‐ and 6‐month time points. Heterogeneity measured by I^‐^squared and Tau‐squared was low for pain and function.

**Conclusion:**

Adding psychological interventions to standard care for knee osteoarthritis improves outcomes in both pain and function. These findings support the integration of psychological interventions into clinical practice.

## 1 Introduction

### 1.1 Background

Osteoarthritis (OA) is a primary cause of disability across the world (Hunter and Bierma‐Zeinstra 2019). The knee is the most common site, accounting for 60% of all known cases of OA (Scheuing et al. 2023). However, knee OA (KOA) cases account for 85% of the total economic burden associated with OA (GBD 2021 Osteoarthritis Collaborators 2023; Scheuing et al. 2023). Additionally, by 2050, cases of KOA are predicted to increase by 75% (GBD 2021 Osteoarthritis Collaborators 2023). In the the United States, an analysis of clinical, quality of life, and economic outcomes estimated that excess healthcare costs for OA patients are $45.4billion annually (Zhao et al. 2019). Also, those with OA and depression have a 39% larger financial healthcare burden compared to patients with OA alone (S. T. Wang and Ni 2022). Existing nonsurgical treatments are ineffective at targeting all contributors to pain (Hunter and Bierma‐Zeinstra 2019). In addition, many patients with KOA do not receive evidence based nonsurgical management strategies (Hunter and Bierma‐Zeinstra 2019).

OA is a condition affecting the articular cartilage, synovium, peri‐articular bone and peri‐articular muscles (Hunter and Bierma‐Zeinstra 2019). There is poor correlation between radiological findings and primary presenting symptoms, such as pain and disability (Culvenor et al. 2019). Pain from KOA can be better explained within the biopsychosocial model which incorporates physical, psychological and social factors (Salaffi et al. 2014). Furthermore, psychological risk factors such as depression, anxiety, fear avoidance beliefs, and pain catastrophising contribute to poor outcomes in those with KOA (Iijima et al. 2018; Palazzo et al. 2016; Phyomaung et al. 2014). Therefore, addressing both physical and psychological aspects of KOA is essential for effective management (Dieppe et al. 2016).

The treatment of psychological aspects of chronic pain has recently drawn increased attention (Williams et al. 2020). Psychological interventions, including cognitive behavioural therapy (CBT) and other CBT based interventions such as pain coping skills training (PCST), aim to modify negative thoughts, emotions and behaviours (Hodges et al. 2011). Both PCST and CBT based interventions have been shown to improve psychological outcomes in patients with KOA (Broderick et al. 2014), however, the effects on physical outcomes are inconclusive (Ismail et al. 2017). Previous systematic reviews have investigated the effects of CBT or PCST on KOA pain (Ismail et al. 2017; Pitsillides et al. 2021), but, to date, no systematic review has examined the impact of combining psychological interventions with standard nonsurgical KOA management.

The aim of this systematic review and meta‐analysis was to evaluate the effectiveness of combining CBT or PCST with standard nonsurgical care on pain and function, as measured by validated pain or functional outcome measures for individuals with KOA.

### 1.2 Objectives

1. To evaluate the effect of adding CBT or PCST to standard care on pain and function in individuals with KOA.

### 1.3 Secondary Objectives

1. To determine whether online, face to face group or face to face individual delivery methods are more effective.
2. To identify if the professional background of the clinicians providing the psychological intervention (CBT or PCST) influences the effectiveness of the intervention.
3. To assess whether CBT or PCST is more effective in improving outcomes.

## 2 Methods

This systematic review was conducted in line with the preferred reporting items for systematic reviews and meta‐analyses (PRISMA) protocol (Page et al. 2021) (Supporting Information S1: Figure S4).

### 2.1 Registration

The systematic review has been registered with the Open Science Framework https://osf.io/k7eg4. The research protocol was submitted to the University of Plymouth.

### 2.2 Search Strategy

A preliminary search of CINAHL, EMBASE (OVID), Medline (EBSCO) and PsycINFO revealed no systematic reviews assessing the effect of adding PCST or CBT to standard care on pain and function.

The full search strategy was designed in collaboration with a library information specialist from The University of Plymouth and peer reviewed by the second reviewer. Randomised controlled trials (RCTs) were identified by searching CINAHL, EMBASE (OVID), Medline (EBSCO) and PsycINFO with additional hand searching of the results.

A PICOS design (Eriksen and Frandsen 2018) was utilised as the search strategy framework (Supporting Information S1: Figure S2.). Wildcards, MeSH terms and Key word searches in the relevant databases were utilised to maximise discoverability. No limit to publication date was applied. The full search details for EMBASE (OVID) are available in the supplementary information (Supporting Information S1: Figure S1). The final search for all databases was completed on July 11, 2024.

### 2.3 Inclusion Criteria

Studies were included if they met the following criteria:

- Adult participants (aged 18 or over) with a clinical or radiological diagnosis of OA, both of which are validated in research and clinical settings (NICE 2022; Q. Wang et al. 2024)
- RCTs with appropriate ethical approval including CBT or PCST combined with standard, routine care and/or exercise as the intervention arm. Previous literature shows that CBT and PCST are the primary psychological interventions investigated in relation to KOA (Tan et al. 2021). Control group interventions could include standard, routine care and/or exercise. Exercise was included as it is a fundamental component of nonsurgical care for KOA (Hunter and Bierma‐Zeinstra 2019). RCTs were included to ensure high quality evidence and support meta‐analysis, improving the potential impact of the current review (Charrois 2015; Sterne et al. 2019)
- Studies using validated tools for evaluating pain or function in KOA to maximise search results.
- Studies in the English language (or able to be translated into the English language).

### 2.4 Exclusion Criteria

- Pilot and feasibility randomised controlled trials were not included as these designs are not powered for efficacy and may bias the results of any analysis (Sterne et al. 2019).
- Studies judged to be at a high risk of bias (see Section 2.5) were excluded from the meta‐analysis in line with recommendations in the Cochrane handbook (Higgins et al. 2019)

### 2.5 Data Extraction and Quality Assessment (Risk of Bias)

Duplicates were identified and removed using Rayyan (Ouzzani et al. 2016). Two reviewers independently screened the title, abstracts and full text articles. The same reviewers extracted data using the Cochrane data extraction tool (Higgins et al. 2019) which was modified to suit the data fields required for the current review. A third reviewer was available to resolve discrepancies; however, this was not needed. The full inclusion and exclusion criteria used to screen articles are detailed in Supporting Information S1: Figure S3.

Risk of bias was independently assessed by two reviewers using the revised Cochrane tool for assessing risk of bias in randomised trials (ROB2) (Sterne et al. 2019). Discrepancies were checked using the ROB2 excel macros, and a third reviewer resolved discrepancies.

### 2.6 Data Analysis

Statistical analysis was performed using Statistical Package for the Social Sciences (SPSS) version 28.0.1.1. All statistical tests were carried out under the guidance of a statistician from the University of Plymouth. Baseline and post‐treatment data were gathered in the form of mean change from baseline for pain and function and standard deviations (SD) at a post‐intervention timepoint that was closest to 6 months. Where mean changes from baseline data were not published, these figures were calculated by subtracting the post‐treatment scores from the baseline scores. Where the SD for the mean change from baseline were not presented, these were imputed using formulas from the Cochrane handbook (Deeks et al. 2023) using either effect sizes or confidence intervals where available. Mean differences (MD) were pooled under a random‐effects model to control for heterogeneity. As studies used different outcomes to evaluate pain and function, standardised mean differences (SMDs) were used to calculate treatment effect sizes from the end of treatment. Effect sizes were calculated using Cohen's d statistic with 95% confidence intervals. *p* values of < 0.05 were considered statistically significant. Effect sizes were assessed as small (0.2), moderate (0.5) or large (> 0.8) (Cohen 1988.).

Heterogeneity was assessed using I squared (I^2^), which was calculated from Q statistic (I^2^ values of 0%–40% indicated low heterogeneity and 30%–60% moderate heterogeneity) and Tau‐squared (a value closest to 0 indicating homogeneity) (Deeks et al. 2023). A funnel plot and Egger's test were not used to assess publication bias, as these are inadequately powered with less than 10 studies (Higgins and Thomas 2023).

## 3 Results

### 3.1 Study Selection

**FIGURE 1:**
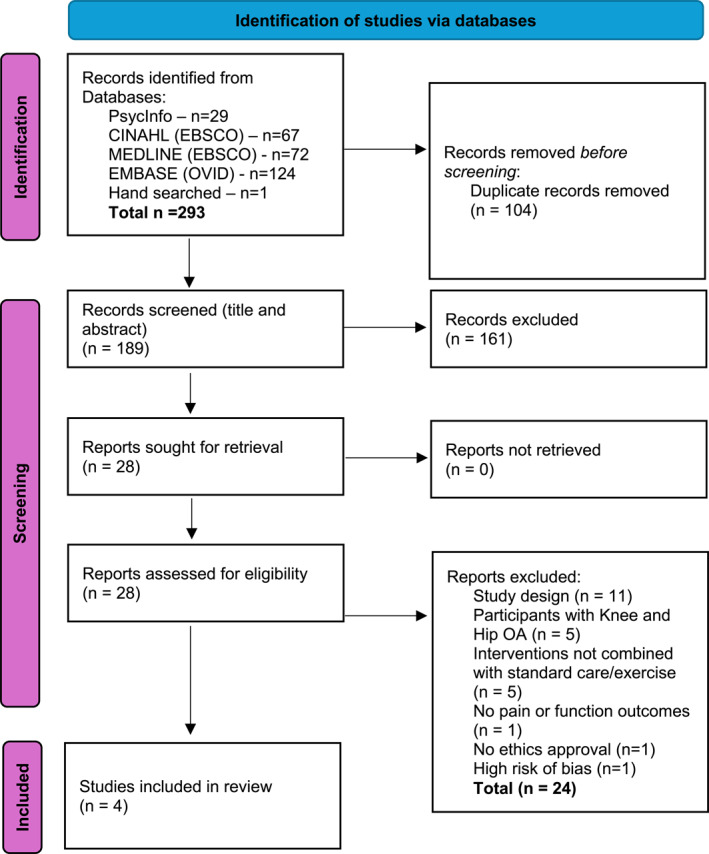
PRISMA study screening process flow diagram.

A total of 189 records were identified after duplicates were removed. Following the PRISMA screening process (Page et al. 2021) (Figure 1), four studies were included in the systematic review. Detailed characteristics of the four included studies are presented in Table 1.

**TABLE 1:** Included study characteristics.

Study	Country	Population	Setting	Intervention	Control	Professional	Mode of delivery	Treatment time	Diagnosis	Primary outcome	Assessment points	Intervention group N =	Mean Age (SD)	Control group N =	Mean Age	SD
Bennell et al. (2016)	Australia	Community adults > 50	Clinic	PCST & exercise	Exercise	Physiotherapist	Individual	12 weeks	ACR	WOMAC pain and function	0, 1, 32, 52 weeks	73	64.6 (8.3)	75	62.7	7.9
Foo et al. (2020)	Malaysia	Community adults 35–75	Clinic	CBT & standard physiotherapy care	Standard physiotherapy care	Physiotherapist and nurses	Group	6 weeks	KL ≥ GII	KOOS pain and function	0, 1, 6 months	150	NA	150	NA	NA
Helminen et al. (2015)	Finland	Community adults 35–75	Primary care	CBT & GP care	GP care	Physiotherapist and psychologist	Group	6 weeks	KL ≥ GII	WOMAC pain and function	0, 3, 12 months	55	64.5 (7.3)	56	62.8	7.2
O’Moore et al. (2018)	Australia	Community adults > 50 with depression	Clinic	CBT & standard care	Standard care	Online programme designed by psychologist	Online self directed	6 online sessions	ACR	WOMAC pain and function	0 months, immediately post treatment, 3 months	44	63.16 (7.4)	25	59.68	6.0

Abbreviations: ACR, American College of Rheumatology; CBT, cognitive behavioural therapy; KL, Kellgren Lawrence; KOOS, knee injury and osteoarthritis outcome score; NA, not available; PCST, pain coping skills training; SD, standard deviation; WOMAC, Western Ontario and McMaster University Osteoarthritis Index.

### 3.2 Study Characteristics

#### 3.2.1 Interventions

Three studies (Foo et al. 2020; Helminen et al. 2015; O’Moore et al. 2018) compared CBT to standard care, while the final study (Bennell et al. 2016) compared PCST to standard care.

#### 3.2.2 Participants

A total of 628 participants were included in the final analysis, 306 of those in the control groups and 322 in the intervention groups. The mean age of the participants in the three studies was 62.9 years (Bennell et al. 2016; Helminen et al. 2015; O’Moore et al. 2018). Foo et al. (2020) presented their age demographics in 10‐year groups with the largest group (38%) aged 56–65, which is consistent with the other three studies.

#### 3.2.3 Diagnostic Criteria

The four studies included in the meta‐analysis used either the Kellgren‐Lawrence osteoarthritis diagnostic criteria (Foo et al. 2020; Helminen et al. 2015) or the American College of Rheumatology criteria (Bennell et al. 2016; O’Moore et al. 2018). These are both validated diagnostic criteria for OA (Q. Wang et al. 2024).

#### 3.2.4 Outcome Measures

Three of the included studies (Bennell et al. 2016; Helminen et al. 2015; O’Moore et al. 2018) used both Western Ontario McMaster University Osteoarthritis Index (WOMAC) pain and function sub sections as outcome measures. Foo et al. (2020) used the Knee injury and osteoarthritis outcome score (KOOS) pain and function sub‐sections.

### 3.3 Risk of Bias

**FIGURE 2:**
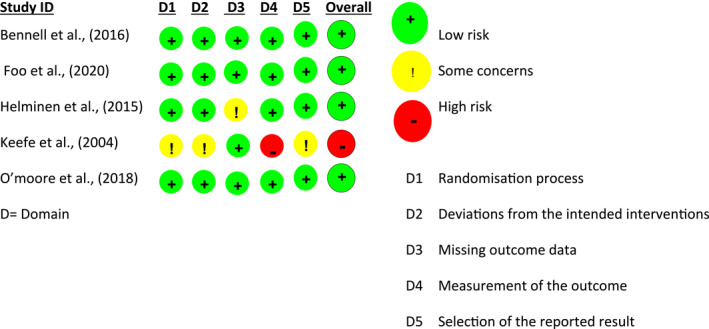
Risk of bias (RoB2) assessment.

**FIGURE 3:**
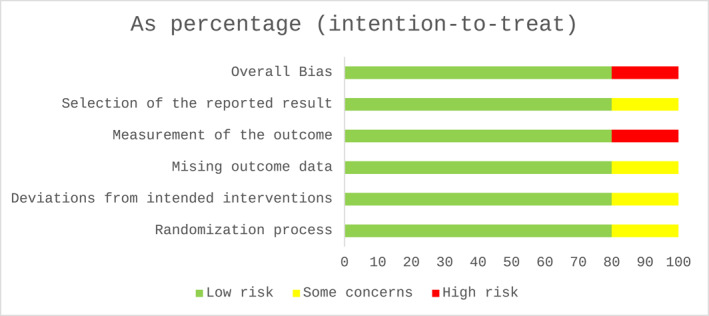
Risk of bias (RoB2) assessment as a percentage.

Risk of bias was assessed using the RoB2 tool (Sterne et al. 2019). Five studies underwent assessment; four (Bennell et al. 2016; Foo et al. 2020; Helminen et al. 2015; O’Moore et al. 2018) were rated as low risk of bias and were included in the final analysis. A fifth study (Keefe et al. 2004) was excluded with an overall rating of high risk of bias because of a high risk of bias judgement in domain four (measurement of the outcome). More detailed results on risk of bias assessment are presented in Figures 2 and 3 and Supporting Information S1: Figure S6.

### 3.4 Pain and Function

**FIGURE 4:**
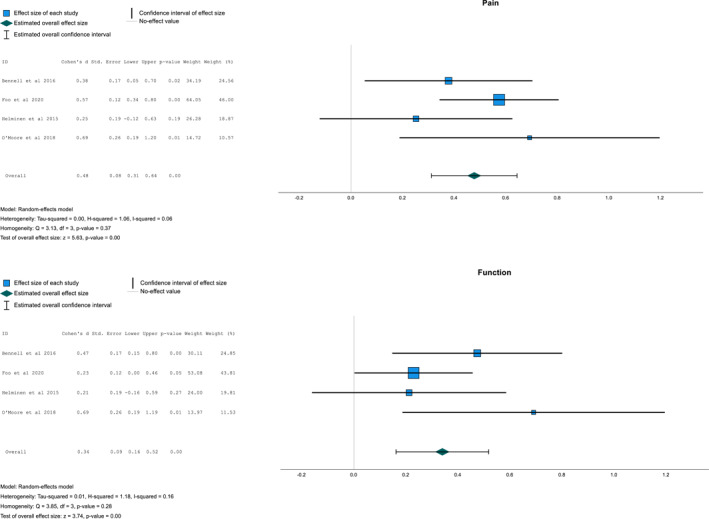
Meta‐analysis forest plot for pain and function.

Pooled results (Figure 4) demonstrated statistically significant improvement in both pain and function outcomes (*P* = < 0.001) favouring intervention. The SMD for pain was 0.48 (95% CI 0.31–0.64) and function was 0.34 (95% CI 0.16–0.52). Using Cohens d statistic, these effect sizes are assessed as small to moderate (Higgins and Thomas 2023).

### 3.5 Heterogeneity

Heterogeneity was assessed using I^2^ test which was calculated from Cochrane's Q statistic. This demonstrated low heterogeneity (0.16) for function and pain outcomes (0.06) (Higgins and Thomas 2023). These findings were further supported by Tau‐squared which indicated low levels of heterogeneity of 0.01 for function and 0.00 for pain outcomes (Higgins 2008).

### 3.6 Secondary Objectives

Statistical analysis of the secondary objectives was not feasible due to the small number of studies included. A narrative review of these secondary objectives was therefore carried out and is presented below.

Secondary objective 1—To determine whether particular delivery methods are more effective. Delivery methods included group (Foo et al. 2020; Helminen et al. 2015), one‐to‐one formats (Bennell et al. 2016) and online (O’Moore et al. 2018). Group delivery by Helminen et al. (2015) reported a small non‐statistically significant effect size for function (0.21) and pain (0.26). In contrast, Foo et al. (2020) reported a statistically significant small effect size for function (0.23) and moderate effect size for pain (0.57). All delivery methods in other studies reported a statistically significant improvement in outcomes in both pain and function.

Secondary objective 2—To identify if the professional background of the clinicians providing the psychological intervention (CBT or PCST) influences the effectiveness of the intervention. While Helminen et al. (2015) reported non‐statistically significant results utilising physiotherapists and psychologists to deliver their psychological intervention, other studies demonstrated effectiveness citing statistically significant results when interventions were delivered by physiotherapists (Bennell et al. 2016; Foo et al. 2020), nurses (Foo et al. 2020) or an online programme developed by psychologists (O’Moore et al. 2018).

Secondary Objective 3—To assess whether CBT or PCST is more effective in improving outcomes. There was insufficient data to assess whether CBT or PCST was more effective as only one included study examined PCST (Bennell et al. 2016). This study showed statistically significant, small to moderate effect sizes for both pain (0.38) and function (0.47).

## 4 Discussion

### 4.1 Primary Objective

This systematic review shows that the addition of CBT or PCST to standard care for KOA improves pain and function outcomes.

This is the first systematic review and meta‐analysis investigating the effects of adding psychological interventions to standard care for KOA on both pain and function. This represents the first step in providing an evidence base to effectively integrate these interventions into clinical practice.

### 4.2 Secondary Objectives

Due to the small number of studies included in the current review, and following consultation with a statistician, a meta‐analysis of secondary objectives was not feasible; therefore, a narrative review was conducted.

The delivery method did not appear to influence the effectiveness of the interventions. This could provide flexibility in delivery to suit the needs of both patients and healthcare providers in practice.

The professional background of the clinicians does not appear to influence the effectiveness of the intervention. These results are supported by Pitsillides et al. (2021) who found that physiotherapist delivered CBT was effective at improving pain outcomes, and Broderick et al. (2014) who demonstrated that nurse practitioners can effectively deliver PCST for those with KOA. This suggests that psychological interventions can be effectively delivered by a range of trained health professionals. Since physiotherapists provide the majority of nonsurgical care for individuals with KOA in the United Kingdom (Smith et al. 2019), providing training for physiotherapists in the delivery of psychological interventions would improve the likelihood of integration into clinical practice in that setting.

### 4.3 Rigour of the Included Studies

The methodological rigour of the included studies was assessed using RoB2. RoB2 consistently highlights problems RCTs face in this field of research, where blinding of the participants and personnel delivering interventions is not possible (Armijo‐Olivo et al. 2017; Juul et al. 2021). One included study (O’Moore et al. 2018) utilised pre‐recorded online delivery of CBT which eliminates the need to blind personnel delivering the intervention and helps to reduce performance bias. Bennell et al. (2016) blinded personnel to the study hypothesis which also helps to reduce performance bias (Viera and Bangdiwala 2007). Blinding of assessors occurred in all the included studies; this reduces the risk of detection bias and improves confidence in the results of the current review (Forbes 2013).

O’Moore et al. (2018) only included individuals with high levels of depression, which could affect the generalisability to individuals with OA that do not have co‐morbid depression. However, the prevalence of depression for individuals with KOA is estimated to be 20% versus 5% in the general population (S. T. Wang and Ni 2022). Therefore, including this cohort of patients with KOA improves external validity for this patient group (Dijkers 2011). Furthermore, there was no difference between reported baseline characteristics for all included studies which improves confidence in the results (Kahan et al. 2015).

Variation in delivery of psychological interventions as, outlined in Table 1, improves external validity as this more accurately reflects real world clinical situations (Findley et al. 2021). Although this could introduce heterogeneity (Findley et al. 2021), it also shows that a range of trained healthcare professionals can deliver these interventions in different settings, which, as previously stated, provides flexibility to both patients and healthcare providers.

Another difference between the studies was the content of standard care controls. Bennell et al. (2016) used an exercise intervention which is a key element of nonsurgical management for KOA (Hunter and Bierma‐Zeinstra 2019). O’Moore et al. (2018) describes ‘treat as usual’ for their control group and Helminen et al. (2015) describes GP care. Neither author provided information on the management to which the control groups were exposed. Participants in Foo et al.’s (2020) control group received standard physiotherapy care including exercise and education. This variation in control groups may be another source of heterogeneity, however this better reflects real world treatment and improves generalisability. Furthermore, all control interventions were then combined with the psychological interventions in the intervention arms of the included studies, this minimises the impact on internal validity and improves confidence in the results (Thompson and Schoenfeld 2007).

Additionally, there were multiple post‐treatment assessment timepoints across the four studies. Two studies (Bennell et al. 2016; Foo et al. 2020) had 6‐month assessment points and the final two studies were at 3 months (Helminen et al. 2015; O’Moore et al. 2018). As the long‐term effects of psychological interventions for KOA are unknown, these varied follow‐up time points may help in assessing effectiveness across different periods. However, this could also introduce heterogeneity which can impact the effect sizes in meta‐analysis (Higgins et al. 2003).

### 4.4 Existing Research

Findings from the meta‐analysis are in line with previous systematic reviews evaluating CBT or PCST on osteoarthritis pain and/or function. Pitsillides et al. (2021) concluded that the addition of CBT to exercise is effective in reducing pain; however, their review has limitations. Pitsillides et al. (2021) included study designs and interventions outside of their search criteria, increasing the risk of evidence selection bias (Drucker et al. 2016). A narrative systematic review by Ismail et al. (Ismail et al. 2017) concluded that more rigorous studies are required to investigate the effect of both CBT and PCST on KOA pain.

Another recent systematic review and meta‐analysis (L. Wang et al. 2021) showed that PCST is effective in reducing pain and function compared with controls. However, in contrast to the current review, L. Wang et al. (2021) were not able to demonstrate similar improvements when PCST was added to standard care. L. Wang et al.'s (2021) review revealed some ethical concerns, such as the inclusion of studies without ethical approval (Keefe et al. 1990). Furthermore, there was a lack of transparency over the statistical analysis which raises concerns around reporting bias (Drucker et al. 2016). However, the authors registered the protocol with Prospero which helps to mitigate this.

Other systematic reviews investigating the effect of psychological interventions in other chronic pain conditions have shown similar results. A systematic review and network meta‐analysis comprising 97 randomised controlled trials and 13,136 participants found that a range of psychological interventions for low back pain were most effective on pain and function when combined with other nonsurgical interventions (Ho et al. 2022). A Cochrane review (Williams et al. 2020) examining the effects of psychological therapies on a variety of chronic pain conditions, including KOA, found small or very small effect sizes for CBT on improving pain, disability and distress. They studied a large evidence base with over 5000 participants; the evidence base studied, however, was rated as low overall quality. These findings in other chronic pain conditions support the results of the current review, demonstrating the effect of adding psychological interventions to standard care for a variety of chronic pain conditions.

### 4.5 Strengths and Limitations

The current review assessed a small number of primary studies in the meta‐analysis which could introduce publication bias (van Aert et al. 2019). Furthermore, using funnel plot and eggers tests to assess for publication bias is not suitable in meta‐analyses of less than 10 studies (Rothstein et al. 2007). However, a visual analysis of the forest plot shows consistent small to moderate effects on both pain and function, across all studies, which helps improve confidence in the results (Brush et al. 2024).

The authors of the current review followed the PRISMA guidelines, and the protocol was submitted to The University of Plymouth; however, it was not registered with Prospero due to Prospero redacting access for MSc projects. However, the current systematic review was registered with the Open Science Framework.

Additionally, there were some changes to the methodology from the protocol; the RoB2 risk of bias tool was chosen over the physiotherapy evidence database scale (PEDro) as PEDro was designed to assess the methodology of physiotherapy trials (Cashin and McAuley 2020). Because of the inclusion of research from varied professional disciplines, the RoB2 tool was selected to improve robustness. RoB2 is the gold standard risk of bias assessment tool for systematic reviews which improves methodological rigour (Higgins et al. 2019). Furthermore, pain outcomes were added as incorporating both function and pain is a better measure of overall disability (Ayis and Dieppe 2009). Decisions were made prior to searches being carried out to minimise the risk of reporting bias.

The review team did not specify a priori what effect sizes would relate to a Minimal Clinically Important Difference (MCID) for knee pain and function. There is a large range of reported MCID values for knee pain and function in the both the WOMAC and the KOOS scales (Angst et al. 2018; Concoff et al. 2019; Denika and Silva 2023). Despite this variability, the size and direction of the pooled SMD in this meta‐analysis increases confidence that these results would be clinically meaningful for patients.

Acknowledging some limitations associated with the small number of included studies, the current review is amongst the most methodologically rigorous meta‐analyses that examine the effect of psychological interventions on KOA.

### 4.6 Future Research

Further research should prioritise large high quality randomised controlled trials to determine the most effective intervention and its delivery method. Additional research should also aim to establish the long‐term effectiveness of psychological interventions for KOA and whether this leads to an improved quality of life and reduction in the economic burden of the condition.

Additionally, future research and consensus on what change represents a MCID in KOA for pain and function using the WOMAC and KOOS would support more robust future meta‐analysis in this field.

### 4.7 Implications for Clinical Practice

This systematic review represents the first step in providing an evidence base to effectively integrate these interventions into clinical practice.

The findings suggest that any trained health professional can effectively provide either CBT or PCST. As physiotherapists provide the majority of nonsurgical care for individuals with KOA (Smith et al. 2019), upskilling physiotherapists in the delivery of psychological interventions would improve integration into clinical practice.

Furthermore, group, one‐to‐one and online delivery all demonstrate effectiveness in improving outcomes for patients with KOA. This could provide flexibility in delivery to suit the needs of both patients and healthcare providers in practice.

## 5 Conclusion

This is the first systematic review and meta‐analysis that demonstrated that the addition of CBT or PCST to standard care for KOA could lead to significantly improved pain and function. These results are valuable to both individuals with KOA and healthcare providers as they could improve the delivery of nonsurgical care and reduce the healthcare and socioeconomic burden of the condition.

## Author Contributions

**Tim Phelps:** Lead Author. **Jonathan Gilby:** Study design, third reviewer and editing for publication. **Dr Joanne Hosking:** Senior Research Fellow, Medical Statistics University of Plymouth, Study design, statistical analysis, editing for publication. **Jonathan Gill:** Study design, data collection, second reviewer and editing for publication.

## Ethics Statement

There are no ethical concerns identified in the current review.

## Conflicts of Interest

The authors declare no conflicts of interest.

## Supporting information

Supporting Information S1

## Data Availability

The authors have nothing to report.
