# Supplementary material for: Psychological Interventions Added to Standard Care Improve Pain and Function Outcomes in Knee Osteoarthritis: A Systematic Review and Meta‐Analysis
Source: Musculoskeletal Care. 2025 Jun 18;23(2):e70141. doi: 10.1002/msc.70141 (PMC12176529; doi:10.1002/msc.70141)
Supplement: Supplementary file 1 — Supporting Information S1 [file MSC-23-e70141-s001.docx]

**Supplementary information**

## **Figure 1. Search strategy**

**Database: Embase <1974 to 2024 July 11>**
**Search Strategy:**
**1**  knee osteoarthritis/ (47457)
**2**  cognitive behavioral therapy/ (28591)
**3**  pain coping skills training.ti,ab. (110)
**4**  knee oa.ti,ab. (15632)
**5**  knee osteoarthr*.ti,ab. (27093)
**6**  degenerative knee arthr*.ti,ab. (32)
**7**  knee arthrosis.ti,ab. (198)
**8**  ((cognitive.mp. adj1 therapy/) or CBT.mp. or cognitive.mp.) adj1 behavio?ral.mp. adj1 therapy/ [mp=title, abstract, heading word, drug trade name, original title, device manufacturer, drug manufacturer, device trade name, keyword heading word, floating subheading word, candidate term word] (2647)
**9**  pcst.ti,ab. (144)
**10**  1 or 4 or 5 or 6 or 7 (52574)
**11**  2 or 3 or 8 or 9 (30463)
**12**  10 and 11 (124)

Figure 2. PICOS search terms

| **Population** | **Intervention** | **Control** | **Outcome** | **Study design** |
| --- | --- | --- | --- | --- |
| Knee osteoarthritis | Cognitive behavio?ral therapy | Standard care | WOMAC  Function sub section | Randomised controlled trials |
| Knee oa | CBT | Control | WOMAC  Pain sub section |  |
| Knee arthrosis | Cognitive therapy | Exercise | Validated function outcome measure |  |
| Knee osteoarth* | Pain coping skills training |  | Validated pain measure |  |
| Degenerative arth* | PCST |  |  |  |
|  | Coping training |  |  |  |

Figure 3. PICOS Inclusion and exclusion criteria

| **PICOS** | **Inclusion** | **Exclusion** |
| --- | --- | --- |
| Population | Knee osteoarthritis diagnosed clinically or with validated diagnostic measure. | No defined osteoarthritis diagnosis.  Previous or planned surgery.  Diagnosis of inflammatory arthritis. |
| Intervention | Pain Coping Skills Training (PCST)  Cognitive behavioural therapy (CBT)  Combined with standard/ routine care or exercise. | No clear description of interventions used.  Other interventions. |
|  | Any duration of intervention will be included. |  |
| Comparator | Standard/routine care.  Exercise. | Other control groups. |
| Outcome | Validated osteoarthritis function outcome measure e.g. WOMAC.  Validated osteoarthritis pain outcome e.g. pain sub section WOMAC. | Pain and or functional outcome measures not validated for use with knee osteoarthritis. |
| Study design | Randomised controlled trials. | Pilot, Cohort, Observational, Case control studies. |
|  | Published research in peer reviewed journals. | High risk of bias from included studies as judged by RoB2 tool assessment. |
|  | No limit to publication date. | No ethics approval gained, or ethics concerns raised during evaluation. |
|  | Written in the English language. |  |

## **Figure 4. PRISMA checklist**

| **Section and Topic** | **Item #** |  | | **Checklist item** | **Location where item is reported** |
| --- | --- | --- | --- | --- | --- |
|  | | | **TITLE** | |  |
| Title | 1 |  | | Identify the report as a systematic review. | 1, 3 |
|  |  |  | |  |  |
|  | | | **ABSTRACT** | |  |
| Abstract | 2 |  | | See the PRISMA 2020 for Abstracts checklist. | 1, Supplementary information |
|  | | | **INTRODUCTION** | |  |
| Rationale | 3 |  | | Describe the rationale for the review in the context of existing knowledge. | 2, 3 |
| Objectives | 4 |  | | Provide an explicit statement of the objective(s) or question(s) the review addresses. | 3 |
|  | | | **METHODS** | |  |
| Eligibility criteria | 5 |  | | Specify the inclusion and exclusion criteria for the review and how studies were grouped for the syntheses. | 3-6 |
| Information sources | 6 |  | | Specify all databases, registers, websites, organisations, reference lists and other sources searched or consulted to identify studies. Specify the date when each source was last searched or consulted. | 4 |
| Search strategy | 7 |  | | Present the full search strategies for all databases, registers and websites, including any filters and limits used. | Supplementary information page 1 |
| Selection process | 8 |  | | Specify the methods used to decide whether a study met the inclusion criteria of the review, including how many reviewers screened each record and each report retrieved, whether they worked independently, and if applicable, details of automation tools used in the process. | 4-6 |
| Data collection process | 9 |  | | Specify the methods used to collect data from reports, including how many reviewers collected data from each report, whether they worked independently, any processes for obtaining or confirming data from study investigators, and if applicable, details of automation tools used in the process. | 4-7 |
| Data items | 10a |  | | List and define all outcomes for which data were sought. Specify whether all results that were compatible with each outcome domain in each study were sought (e.g. for all measures, time points, analyses), and if not, the methods used to decide which results to collect. | 4-6 |
|  | 10b |  | | List and define all other variables for which data were sought (e.g. participant and intervention characteristics, funding sources). Describe any assumptions made about any missing or unclear information. | 4-6 |
| Study risk of bias assessment | 11 |  | | Specify the methods used to assess risk of bias in the included studies, including details of the tool(s) used, how many reviewers assessed each study and whether they worked independently, and if applicable, details of automation tools used in the process. | 5 |
| Effect measures | 12 |  | | Specify for each outcome the effect measure(s) (e.g. risk ratio, mean difference) used in the synthesis or presentation of results. | 6 |
| Synthesis methods | 13a |  | | Describe the processes used to decide which studies were eligible for each synthesis (e.g. tabulating the study intervention characteristics and comparing against the planned groups for each synthesis (item #5)). | 6 |
|  | 13b |  | | Describe any methods required to prepare the data for presentation or synthesis, such as handling of missing summary statistics, or data conversions. | 6 |
|  | 13c |  | | Describe any methods used to tabulate or visually display results of individual studies and syntheses. | 6 |
|  | 13d |  | | Describe any methods used to synthesize results and provide a rationale for the choice(s). If meta-analysis was performed, describe the model(s), method(s) to identify the presence and extent of statistical heterogeneity, and software package(s) used. | 6 |
|  | 13e |  | | Describe any methods used to explore possible causes of heterogeneity among study results (e.g. subgroup analysis, meta-regression). | 6 |
|  | 13f |  | | Describe any sensitivity analyses conducted to assess robustness of the synthesized results. | 6 |
| Reporting bias assessment | 14 |  | | Describe any methods used to assess risk of bias due to missing results in a synthesis (arising from reporting biases). | 6 |
| Certainty assessment | 15 |  | | Describe any methods used to assess certainty (or confidence) in the body of evidence for an outcome. | 6 |
|  | | | **RESULTS** | |  |
| Study selection | 16a |  | | Describe the results of the search and selection process, from the number of records identified in the search to the number of studies included in the review, ideally using a flow diagram. | 6-10 |
|  | 16b |  | | Cite studies that might appear to meet the inclusion criteria, but which were excluded, and explain why they were excluded. | 8 |
| Study characteristics | 17 |  | | Cite each included study and present its characteristics. | 7,8,12 |
| Risk of bias in studies | 18 |  | | Present assessments of risk of bias for each included study. | 8,13 |
| Results of individual studies | 19 |  | | For all outcomes, present, for each study: (a) summary statistics for each group (where appropriate) and (b) an effect estimate and its precision (e.g. confidence/credible interval), ideally using structured tables or plots. | 9,11,12 |
| Results of syntheses | 20a |  | | For each synthesis, briefly summarise the characteristics and risk of bias among contributing studies. | 8-10,13 |
|  | 20b |  | | Present results of all statistical syntheses conducted. If meta-analysis was done, present for each the summary estimate and its precision (e.g. confidence/credible interval) and measures of statistical heterogeneity. If comparing groups, describe the direction of the effect. | 9, 12 |
|  | 20c |  | | Present results of all investigations of possible causes of heterogeneity among study results. | 8-10, 12 |
|  | 20d |  | | Present results of all sensitivity analyses conducted to assess the robustness of the synthesized results. | 9,12 |
| Reporting biases | 21 |  | | Present assessments of risk of bias due to missing results (arising from reporting biases) for each synthesis assessed. | 13 |
| Certainty of evidence | 22 |  | | Present assessments of certainty (or confidence) in the body of evidence for each outcome assessed. | 9, 12 |
|  | | | **DISCUSSION** | |  |
| Discussion | 23a |  | | Provide a general interpretation of the results in the context of other evidence. | 14 |
|  | 23b |  | | Discuss any limitations of the evidence included in the review. | 17,18 |
|  | 23c |  | | Discuss any limitations of the review processes used. | 17,18 |
|  | 23d |  | | Discuss implications of the results for practice, policy, and future research. | 14,18,19 |
|  | | | **OTHER INFORMATION** | |  |
| Registration and protocol | 24a |  | | Provide registration information for the review, including register name and registration number, or state that the review was not registered. | 4 |
|  | 24b |  | | Indicate where the review protocol can be accessed, or state that a protocol was not prepared. | 4 |
|  | 24c |  | | Describe and explain any amendments to information provided at registration or in the protocol. | 17, 18 |
| Support | 25 |  | | Describe sources of financial or non-financial support for the review, and the role of the funders or sponsors in the review. | Title page |
| Competing interests | 26 |  | | Declare any competing interests of review authors. | Title page |
| Availability of data, code and other materials | 27 |  | | Report which of the following are publicly available and where they can be found: template data collection forms; data extracted from included studies; data used for all analyses; analytic code; any other materials used in the review. | Supplementary information |

## **Figure 5. PRISMA Abstract checklist**

| **Section and Topic** | **Item #** | **Checklist item** | **Reported (Yes/No)** |
| --- | --- | --- | --- |
| **TITLE** | | |  |
| Title | 1 | Identify the report as a systematic review. | Yes |
| **BACKGROUND** | | |  |
| Objectives | 2 | Provide an explicit statement of the main objective(s) or question(s) the review addresses. | Yes |
| **METHODS** | | |  |
| Eligibility criteria | 3 | Specify the inclusion and exclusion criteria for the review. | No – not in line with journal guidelines |
| Information sources | 4 | Specify the information sources (e.g. databases, registers) used to identify studies and the date when each was last searched. | Yes |
| Risk of bias | 5 | Specify the methods used to assess risk of bias in the included studies. | Yes |
| Synthesis of results | 6 | Specify the methods used to present and synthesise results. | Yes |
| **RESULTS** | | |  |
| Included studies | 7 | Give the total number of included studies and participants and summarise relevant characteristics of studies. | Yes |
| Synthesis of results | 8 | Present results for main outcomes, preferably indicating the number of included studies and participants for each. If meta-analysis was done, report the summary estimate and confidence/credible interval. If comparing groups, indicate the direction of the effect (i.e. which group is favoured). | Yes |
| **DISCUSSION** | | |  |
| Limitations of evidence | 9 | Provide a brief summary of the limitations of the evidence included in the review (e.g. study risk of bias, inconsistency and imprecision). | Yes |
| Interpretation | 10 | Provide a general interpretation of the results and important implications. | Yes |
| **OTHER** | | |  |
| Funding | 11 | Specify the primary source of funding for the review. | No – not in line with journal guidelines |
| Registration | 12 | Provide the register name and registration number. | No – not in line with journal guidelines |

## **Figure 6. Risk of bias 2 tool assessment**

| **Unique ID** | 4 | **Study ID** | 4 | **Assessor** |  |
| --- | --- | --- | --- | --- | --- |
| **Ref or Label** | Bennell et al 2016 | **Aim** | assignment to intervention (the 'intention-to-treat' effect) |  |  |
| **Experimental** | Int | **Comparator** | cont. | **Source** | Journal article(s); Trial protocol |
| **Outcome** | all | **Results** |  | **Weight** | 1 |
| **Domain** | **Signalling question** | | | **Response** | **Comments** |
| **Bias arising from the randomization process** | 1.1 Was the allocation sequence random? | | | Y | Allocation randomised and concealed |
|  | 1.2 Was the allocation sequence concealed until participants were enrolled and assigned to interventions? | | | Y |  |
|  | 1.3 Did baseline differences between intervention groups suggest a problem with the randomization process? | | | N | No differences detected |
|  | **Risk of bias judgement** | | | **Low** |  |
| **Bias due to deviations from intended interventions** | 2.1.Were participants aware of their assigned intervention during the trial? | | | Y | Participants and persons delivering interventions were not able to be blinded due to the nature of the interventions |
|  | 2.2.Were carers and people delivering the interventions aware of participants' assigned intervention during the trial? | | | Y |  |
|  | 2.3. If Y/PY/NI to 2.1 or 2.2: Were there deviations from the intended intervention that arose because of the experimental context? | | | PN | Participants were blinded to the study hypothesis to minimise performance bias |
|  | 2.4 If Y/PY to 2.3: Were these deviations likely to have affected the outcome? | | | NA |  |
|  | 2.5. If Y/PY/NI to 2.4: Were these deviations from intended intervention balanced between groups? | | | NA |  |
|  | 2.6 Was an appropriate analysis used to estimate the effect of assignment to intervention? | | | Y | ITT |
|  | 2.7 If N/PN/NI to 2.6: Was there potential for a substantial impact (on the result) of the failure to analyse participants in the group to which they were randomized? | | | NA |  |
|  | **Risk of bias judgement** | | | **Low** |  |
| **Bias due to missing outcome data** | 3.1 Were data for this outcome available for all, or nearly all, participants randomized? | | | PN | Drop out rate 17% is expected for this type of study, Baseline characteristics are available for all participants. |
|  | 3.2 If N/PN/NI to 3.1: Is there evidence that result was not biased by missing outcome data? | | | PY | Those lost to follow up had less severe disease and showed higher levels of function in baseline outcome measures, meaning this is less likely to affect the outcome data |
|  | 3.3 If N/PN to 3.2: Could missingness in the outcome depend on its true value? | | | NA |  |
|  | 3.4 If Y/PY/NI to 3.3: Is it likely that missingness in the outcome depended on its true value? | | | NA |  |
|  | **Risk of bias judgement** | | | **Low** |  |
| **Bias in measurement of the outcome** | 4.1 Was the method of measuring the outcome inappropriate? | | | N | WOMAC and VAS validated measures |
|  | 4.2 Could measurement or ascertainment of the outcome have differed between intervention groups? | | | N | Assessment protocols identical |
|  | 4.3 Were outcome assessors aware of the intervention received by study participants? | | | N | Assessor blinded |
|  | 4.4 If Y/PY/NI to 4.3: Could assessment of the outcome have been influenced by knowledge of intervention received? | | | NA |  |
|  | 4.5 If Y/PY/NI to 4.4: Is it likely that assessment of the outcome was influenced by knowledge of intervention received? | | | NA |  |
|  | **Risk of bias judgement** | | | **Low** |  |
| **Bias in selection of the reported result** | 5.1 Were the data that produced this result analysed in accordance with a pre-specified analysis plan that was finalized before unblinded outcome data were available for analysis? | | | Y | Outcome measures described in feasibility study Hunt et al 2013 |
|  | 5.2 ... multiple eligible outcome measurements (e.g. scales, definitions, time points) within the outcome domain? | | | N | All outcomes presented |
|  | 5.3 ... multiple eligible analyses of the data? | | | N | No evidence |
|  | **Risk of bias judgement** | | | **Low** |  |
| **Overall bias** | **Risk of bias judgement** | | | **Low** |  |
|  |  |  |  |  |  |
|  |  |  |  |  |  |
| **Unique ID** | 3 | **Study ID** | 3 | **Assessor** |  |
| **Ref or Label** | Foo 2020 | **Aim** | assignment to intervention (the 'intention-to-treat' effect) |  |  |
| **Experimental** | Int | **Comparator** | Cont. | **Source** | Journal article(s) |
| **Outcome** | All | **Results** |  | **Weight** | 1 |
| **Domain** | **Signalling question** | | | **Response** | **Comments** |
| **Bias arising from the randomization process** | 1.1 Was the allocation sequence random? | | | Y | Random allocation process, concealment carried out |
|  | 1.2 Was the allocation sequence concealed until participants were enrolled and assigned to interventions? | | | PY |  |
|  | 1.3 Did baseline differences between intervention groups suggest a problem with the randomization process? | | | N | No differences - this is published in previous research (Foo et al 2017) alluded to in this article |
|  | **Risk of bias judgement** | | | **Low** |  |
| **Bias due to deviations from intended interventions** | 2.1.Were participants aware of their assigned intervention during the trial? | | | Y | Unable to blind participants or those delivering interventions due to the nature of the interventions |
|  | 2.2.Were carers and people delivering the interventions aware of participants' assigned intervention during the trial? | | | Y |  |
|  | 2.3. If Y/PY/NI to 2.1 or 2.2: Were there deviations from the intended intervention that arose because of the experimental context? | | | PN | Nothing identified |
|  | 2.4 If Y/PY to 2.3: Were these deviations likely to have affected the outcome? | | | NA |  |
|  | 2.5. If Y/PY/NI to 2.4: Were these deviations from intended intervention balanced between groups? | | | NA |  |
|  | 2.6 Was an appropriate analysis used to estimate the effect of assignment to intervention? | | | Y | ITT |
|  | 2.7 If N/PN/NI to 2.6: Was there potential for a substantial impact (on the result) of the failure to analyse participants in the group to which they were randomized? | | | NA |  |
|  | **Risk of bias judgement** | | | **Low** |  |
| **Bias due to missing outcome data** | 3.1 Were data for this outcome available for all, or nearly all, participants randomized? | | | PN | 76.67% completed the study |
|  | 3.2 If N/PN/NI to 3.1: Is there evidence that result was not biased by missing outcome data? | | | PY | Similar drop out rates and reasons for drop outs similar   ITT analysis |
|  | 3.3 If N/PN to 3.2: Could missingness in the outcome depend on its true value? | | | NA |  |
|  | 3.4 If Y/PY/NI to 3.3: Is it likely that missingness in the outcome depended on its true value? | | | NA |  |
|  | **Risk of bias judgement** | | | **Low** |  |
| **Bias in measurement of the outcome** | 4.1 Was the method of measuring the outcome inappropriate? | | | PN | KOOS is validated outcome measure |
|  | 4.2 Could measurement or ascertainment of the outcome have differed between intervention groups? | | | N | Identical assessment protocols |
|  | 4.3 Were outcome assessors aware of the intervention received by study participants? | | | N | Blinded assessors |
|  | 4.4 If Y/PY/NI to 4.3: Could assessment of the outcome have been influenced by knowledge of intervention received? | | | NA |  |
|  | 4.5 If Y/PY/NI to 4.4: Is it likely that assessment of the outcome was influenced by knowledge of intervention received? | | | NA |  |
|  | **Risk of bias judgement** | | | **Low** |  |
| **Bias in selection of the reported result** | 5.1 Were the data that produced this result analysed in accordance with a pre-specified analysis plan that was finalized before unblinded outcome data were available for analysis? | | | PY | Previous trial (Foo et al 2017) outlined primary outcome KOOS pain and then KOOS function |
|  | 5.2 ... multiple eligible outcome measurements (e.g. scales, definitions, time points) within the outcome domain? | | | PN | Single measures for outcomes assessed |
|  | 5.3 ... multiple eligible analyses of the data? | | | PN | No evidence |
|  | **Risk of bias judgement** | | | **Low** |  |
| **Overall bias** | **Risk of bias judgement** | | | **Low** |  |
|  |  |  |  |  |  |
|  |  |  |  |  |  |
| **Unique ID** | 2 | **Study ID** | 2 | **Assessor** |  |
| **Ref or Label** | Helminen 2015 | **Aim** | assignment to intervention (the 'intention-to-treat' effect) |  |  |
| **Experimental** | Int | **Comparator** | Cont. | **Source** | Journal article(s); Trial protocol |
| **Outcome** | All | **Results** |  | **Weight** | 1 |
| **Domain** | **Signalling question** | | | **Response** | **Comments** |
| **Bias arising from the randomization process** | 1.1 Was the allocation sequence random? | | | Y | Random allocation  Allocation concealed |
|  | 1.2 Was the allocation sequence concealed until participants were enrolled and assigned to interventions? | | | Y |  |
|  | 1.3 Did baseline differences between intervention groups suggest a problem with the randomization process? | | | N | No differences identified |
|  | **Risk of bias judgement** | | | **Low** |  |
| **Bias due to deviations from intended interventions** | 2.1.Were participants aware of their assigned intervention during the trial? | | | Y | Unable to blind participants or those delivering interventions due to the nature of interventions |
|  | 2.2.Were carers and people delivering the interventions aware of participants' assigned intervention during the trial? | | | Y |  |
|  | 2.3. If Y/PY/NI to 2.1 or 2.2: Were there deviations from the intended intervention that arose because of the experimental context? | | | PN | Nothing identified |
|  | 2.4 If Y/PY to 2.3: Were these deviations likely to have affected the outcome? | | | NA |  |
|  | 2.5. If Y/PY/NI to 2.4: Were these deviations from intended intervention balanced between groups? | | | NA |  |
|  | 2.6 Was an appropriate analysis used to estimate the effect of assignment to intervention? | | | Y | ITT |
|  | 2.7 If N/PN/NI to 2.6: Was there potential for a substantial impact (on the result) of the failure to analyse participants in the group to which they were randomized? | | | NA |  |
|  | **Risk of bias judgement** | | | **Low** |  |
| **Bias due to missing outcome data** | 3.1 Were data for this outcome available for all, or nearly all, participants randomized? | | | N | Higher drop out rates in control 20% vs 4% |
|  | 3.2 If N/PN/NI to 3.1: Is there evidence that result was not biased by missing outcome data? | | | N | Higher levels of TKR 4 vs 1   Dropped our due to dissatisfaction 1 vs 0  Dropped out no response 8 vs 1 |
|  | 3.3 If N/PN to 3.2: Could missingness in the outcome depend on its true value? | | | PY | Due to higher conversion to TKR and dissatisfaction it is likely these participants had higher levels of pain and disability |
|  | 3.4 If Y/PY/NI to 3.3: Is it likely that missingness in the outcome depended on its true value? | | | PN |  |
|  | **Risk of bias judgement** | | | **Some concerns** |  |
| **Bias in measurement of the outcome** | 4.1 Was the method of measuring the outcome inappropriate? | | | N | WOMAC is recommended for use of assessing pain and function outcomes in OA research |
|  | 4.2 Could measurement or ascertainment of the outcome have differed between intervention groups? | | | PN | Identical assessment protocol |
|  | 4.3 Were outcome assessors aware of the intervention received by study participants? | | | N | Blinded assessors |
|  | 4.4 If Y/PY/NI to 4.3: Could assessment of the outcome have been influenced by knowledge of intervention received? | | | NA |  |
|  | 4.5 If Y/PY/NI to 4.4: Is it likely that assessment of the outcome was influenced by knowledge of intervention received? | | | NA |  |
|  | **Risk of bias judgement** | | | **Low** |  |
| **Bias in selection of the reported result** | 5.1 Were the data that produced this result analysed in accordance with a pre-specified analysis plan that was finalized before unblinded outcome data were available for analysis? | | | PY | protocol |
|  | 5.2 ... multiple eligible outcome measurements (e.g. scales, definitions, time points) within the outcome domain? | | | PN | Single measures of outcomes |
|  | 5.3 ... multiple eligible analyses of the data? | | | PN | No evidence |
|  | **Risk of bias judgement** | | | **Low** |  |
| **Overall bias** | **Risk of bias judgement** | | | **Low** |  |
|  |  |  |  |  |  |
|  |  |  |  |  |  |
| **Unique ID** | 5 | **Study ID** | 5 | **Assessor** |  |
| **Ref or Label** | Keefe et al 2004 | **Aim** | assignment to intervention (the 'intention-to-treat' effect) |  |  |
| **Experimental** | Int | **Comparator** | Cont. | **Source** | Journal article(s) |
| **Outcome** | All | **Results** |  | **Weight** | 1 |
| **Domain** | **Signalling question** | | | **Response** | **Comments** |
| **Bias arising from the randomization process** | 1.1 Was the allocation sequence random? | | | Y | Random allocation but no information surrounding allocation concealment |
|  | 1.2 Was the allocation sequence concealed until participants were enrolled and assigned to interventions? | | | NI |  |
|  | 1.3 Did baseline differences between intervention groups suggest a problem with the randomization process? | | | N | No significant differences in baseline groups |
|  | **Risk of bias judgement** | | | **Some concerns** |  |
| **Bias due to deviations from intended interventions** | 2.1.Were participants aware of their assigned intervention during the trial? | | | Y | Unable to blind participants or those delivering intervention due to nature of intervention |
|  | 2.2.Were carers and people delivering the interventions aware of participants' assigned intervention during the trial? | | | Y |  |
|  | 2.3. If Y/PY/NI to 2.1 or 2.2: Were there deviations from the intended intervention that arose because of the experimental context? | | | NI | No information |
|  | 2.4 If Y/PY to 2.3: Were these deviations likely to have affected the outcome? | | | NA |  |
|  | 2.5. If Y/PY/NI to 2.4: Were these deviations from intended intervention balanced between groups? | | | NA |  |
|  | 2.6 Was an appropriate analysis used to estimate the effect of assignment to intervention? | | | PN | ITT not used |
|  | 2.7 If N/PN/NI to 2.6: Was there potential for a substantial impact (on the result) of the failure to analyse participants in the group to which they were randomized? | | | PN | No significant difference in drop out or reasons for drop out. No differences in baseline groups |
|  | **Risk of bias judgement** | | | **Some concerns** |  |
| **Bias due to missing outcome data** | 3.1 Were data for this outcome available for all, or nearly all, participants randomized? | | | Y | 93.1% of all participants completed study |
|  | 3.2 If N/PN/NI to 3.1: Is there evidence that result was not biased by missing outcome data? | | | NA |  |
|  | 3.3 If N/PN to 3.2: Could missingness in the outcome depend on its true value? | | | NA |  |
|  | 3.4 If Y/PY/NI to 3.3: Is it likely that missingness in the outcome depended on its true value? | | | NA |  |
|  | **Risk of bias judgement** | | | **Low** |  |
| **Bias in measurement of the outcome** | 4.1 Was the method of measuring the outcome inappropriate? | | | N | AIMS and VAS validated outcome measure |
|  | 4.2 Could measurement or ascertainment of the outcome have differed between intervention groups? | | | N | Outcome assessment procedure identical |
|  | 4.3 Were outcome assessors aware of the intervention received by study participants? | | | NI | No information |
|  | 4.4 If Y/PY/NI to 4.3: Could assessment of the outcome have been influenced by knowledge of intervention received? | | | NI | No information |
|  | 4.5 If Y/PY/NI to 4.4: Is it likely that assessment of the outcome was influenced by knowledge of intervention received? | | | NI |  |
|  | **Risk of bias judgement** | | | **High** |  |
| **Bias in selection of the reported result** | 5.1 Were the data that produced this result analysed in accordance with a pre-specified analysis plan that was finalized before unblinded outcome data were available for analysis? | | | NI | No information |
|  | 5.2 ... multiple eligible outcome measurements (e.g. scales, definitions, time points) within the outcome domain? | | | N | Single measures for outcomes assessed |
|  | 5.3 ... multiple eligible analyses of the data? | | | N | no evidence of this |
|  | **Risk of bias judgement** | | | **Some concerns** |  |
| **Overall bias** | **Risk of bias judgement** | | | **High** |  |
|  |  |  |  |  |  |
|  |  |  |  |  |  |
| **Unique ID** | 1 | **Study ID** | 1 | **Assessor** |  |
| **Ref or Label** | OMoore 2018 | **Aim** | assignment to intervention (the 'intention-to-treat' effect) |  |  |
| **Experimental** | Int | **Comparator** | cont | **Source** | Journal article(s); Trial protocol |
| **Outcome** | All | **Results** |  | **Weight** | 1 |
| **Domain** | **Signalling question** | | | **Response** | **Comments** |
| **Bias arising from the randomization process** | 1.1 Was the allocation sequence random? | | | Y | Allocation was randomised and allocation concealed |
|  | 1.2 Was the allocation sequence concealed until participants were enrolled and assigned to interventions? | | | Y |  |
|  | 1.3 Did baseline differences between intervention groups suggest a problem with the randomization process? | | | PY | Groups unequal 44 intervention vs 25 control |
|  | **Risk of bias judgement** | | | **Low** |  |
| **Bias due to deviations from intended interventions** | 2.1.Were participants aware of their assigned intervention during the trial? | | | Y | Unable to blind participants and those delivering interventions due to nature of interventions |
|  | 2.2.Were carers and people delivering the interventions aware of participants' assigned intervention during the trial? | | | Y |  |
|  | 2.3. If Y/PY/NI to 2.1 or 2.2: Were there deviations from the intended intervention that arose because of the experimental context? | | | PN | Nothing identified |
|  | 2.4 If Y/PY to 2.3: Were these deviations likely to have affected the outcome? | | | NA |  |
|  | 2.5. If Y/PY/NI to 2.4: Were these deviations from intended intervention balanced between groups? | | | NA |  |
|  | 2.6 Was an appropriate analysis used to estimate the effect of assignment to intervention? | | | Y | ITT analysis |
|  | 2.7 If N/PN/NI to 2.6: Was there potential for a substantial impact (on the result) of the failure to analyse participants in the group to which they were randomized? | | | NA |  |
|  | **Risk of bias judgement** | | | **Low** |  |
| **Bias due to missing outcome data** | 3.1 Were data for this outcome available for all, or nearly all, participants randomized? | | | PN | Missingness for 1 participant in control and 2 participants in interventions group   ITT analysis used |
|  | 3.2 If N/PN/NI to 3.1: Is there evidence that result was not biased by missing outcome data? | | | N | MCAR test showed missing data was at random |
|  | 3.3 If N/PN to 3.2: Could missingness in the outcome depend on its true value? | | | PY | MCAR test showed missing data was at random |
|  | 3.4 If Y/PY/NI to 3.3: Is it likely that missingness in the outcome depended on its true value? | | | PN |  |
|  | **Risk of bias judgement** | | | **Low** |  |
| **Bias in measurement of the outcome** | 4.1 Was the method of measuring the outcome inappropriate? | | | N | WOMAC is recommended for use of assessing pain and function outcomes in OA research |
|  | 4.2 Could measurement or ascertainment of the outcome have differed between intervention groups? | | | N | Nothing detected |
|  | 4.3 Were outcome assessors aware of the intervention received by study participants? | | | N | Assessors blinded |
|  | 4.4 If Y/PY/NI to 4.3: Could assessment of the outcome have been influenced by knowledge of intervention received? | | | NA |  |
|  | 4.5 If Y/PY/NI to 4.4: Is it likely that assessment of the outcome was influenced by knowledge of intervention received? | | | NA |  |
|  | **Risk of bias judgement** | | | **Low** |  |
| **Bias in selection of the reported result** | 5.1 Were the data that produced this result analysed in accordance with a pre-specified analysis plan that was finalized before unblinded outcome data were available for analysis? | | | Y | Protocol - outcome measures and time point clearly defined   Only difference is changing ANOVA to linear mixed methods model which in my mind does not increase bias |
|  | 5.2 ... multiple eligible outcome measurements (e.g. scales, definitions, time points) within the outcome domain? | | | PN | No evidence of this |
|  | 5.3 ... multiple eligible analyses of the data? | | | PN | No evidence of this |
|  | **Risk of bias judgement** | | | **Low** |  |
| **Overall bias** | **Risk of bias judgement** | | | **Low** |  |
